# Subcellular progression of mesenchymal transition identified by two discrete synchronous cell lines derived from the same glioblastoma

**DOI:** 10.1007/s00018-022-04188-3

**Published:** 2022-03-12

**Authors:** Sojin Kim, Soo-Ji Park, Tamrin Chowdhury, Jeong-Im Hong, Jinhee Ahn, Tae Yeong Jeong, Hyeon Jong Yu, Young-Kyoung Shin, Ja-Lok Ku, Jong Bae Park, Junho K. Hur, Hwajin Lee, Kyoungmi Kim, Chul‑Kee Park

**Affiliations:** 1 Department of Neurosurgery, Seoul National University College of Medicine, Seoul National University Hospital, Seoul, 03080 Republic of Korea; 2 Department of Biomedical Laboratory Science, Daegu Health College, Daegu, 41453 Republic of Korea; 3 Department of Physiology, Korea University College of Medicine, Seoul, 02841 Republic of Korea; 4 Department of Biomedical Sciences, Korea University College of Medicine, Seoul, 02841 Republic of Korea; 5 Korean Cell Line Bank, Laboratory of Cell Biology, Cancer Research Institute, Seoul National University College of Medicine, Seoul, 03080 Republic of Korea; 6 Department of Cancer Biomedical Science, Graduate School of Cancer Science and Policy, National Cancer Center, Goyang, 10408 Republic of Korea; 7 Department of Genetics, College of Medicine, Hanyang University, Seoul, 04763 Republic of Korea; 8 Biomedical Knowledge Engineering Laboratory, Seoul National University, Seoul, 08826 Republic of Korea; 9 Genomic Medicine Institute, Medical Research Center, Seoul National University College of Medicine, Seoul, 03080 Republic of Korea

**Keywords:** Glioblastoma, Intratumoral heterogeneity, Mesenchymal transition, Synchronous cell lines

## Abstract

Glioblastomas (GBM) exhibit intratumoral heterogeneity of various oncogenic evolutional processes. We have successfully isolated and established two distinct cancer cell lines with different morphological and biological characteristics that were derived from the same tissue sample of a GBM. When we compared their genomic and transcriptomic characteristics, each cell line harbored distinct mutation clusters while sharing core driver mutations. Transcriptomic analysis revealed that one cell line was undergoing a mesenchymal transition process, unlike the other cell line. Furthermore, we could identify four tumor samples containing our cell line-like clusters from the publicly available single-cell RNA-seq data, and in a set of paired longitudinal GBM samples, we could confirm three pairs where the recurrent sample was enriched in the genes specific to our cell line undergoing mesenchymal transition. The present study provides direct evidence and a valuable source for investigating the ongoing process of subcellular mesenchymal transition in GBM, which has prognostic and therapeutic implications.

**Supplementary Information:**

The online version contains supplementary material available at 10.1007/s00018-022-04188-3.

## Introduction

Glioblastoma (GBM) is the most common primary malignant brain tumor and is one of the most aggressive cancers [1]. Despite the current best practice of maximal safe surgery, chemotherapy, and radiotherapy, the outcome of GBM is still devastating, in part due to intratumoral heterogeneity. Intratumoral heterogeneity implies spatially dispersed genetically diverse populations of cancer cells, distinguishing themselves in a variety of biological functions, including cell proliferation, invasion, immunity, and metastasis. Phylogenic clonal evolution giving rise to differential cellular hierarchies during oncogenesis usually leads to intratumoral heterogeneity in human GBMs. Therefore, intratumoral heterogeneity can contribute to treatment resistance or immune evasion [2].

Epithelial to mesenchymal transition, an EMT process, is a highly controversial term in GBM tumorigenesis. In gliomagenesis and its progression, a non-classical EMT-like or glial to mesenchymal transition is proposed to be linked to the mesenchymal nature of the neural cells during the neurodevelopmental process [3]. Another study reported the onco-plasticity of EMT-like or mesenchymal to epithelial transition (MET)-like conversion mediated by the epigenetic changes in the tumor microenvironment around glioma stem cells [4]. The well-known classification of glioma subtypes by gene expression profiles also uses several EMT-related genes (CD44, MERTK, TGFB1, NOTCH2 etc.), which are enriched in the mesenchymal subtypes [5, 6]. A recent study attempted to classify the GBMs using a series of EMT-associated genes to predict the differences in prognosis [7]. In addition, EMT has been widely described as a key factor that drives cellular heterogeneity influenced by both genetic and non-genetic determinants and even a small number of cells undergoing this process can cause evident intratumoral heterogeneity in cancers [8, 9]. However, the role of EMT and its clinical significance in GBM is still not well established due to the lack of direct evidence of its evolution in clinical samples and the rarity of cell lines that are derived from the same tissue to perform the verifying experiments.

Here, we isolated and established two distinct cell lines derived from the same GBM tissue sample. These cell lines have genetically developed into different cell lines by subculture. We compared and analyzed the genomic and biological signatures of the two cell lines and found that one of the cell lines exhibits mesenchymal transition characteristics, unlike the other. Furthermore, we reanalyzed publicly available single-cell RNA-seq data to identify analogous clusters to our case and found four similar GBM samples with occult clones of mesenchymal transition. We also found recurrent GBM samples showing evolution of mesenchymal transition clones characterized by upregulation of the EMT genes identical to the cell line which was not identified in the initial sample. These findings provide direct evidence to support the subcellular evolution of mesenchymal transition in GBM, which can be utilized in future investigations into intratumoral heterogeneity and the mesenchymal transition process.

## Materials and methods

### Cell lines and culture condition

An IRB-approved written informed consent was obtained from the patient to use the samples and to establish cell lines for research purposes (Seoul National University Hospital IRB No. H-0507-509-153 and H-1102-098-357). Tissues were freshly frozen in liquid nitrogen immediately after resection, and white blood cells (WBCs) were extracted from whole blood. Both tissue and WBC samples were then stored at − 80 °C until later use. The cell lines were established using the core area of the fresh tumor tissue just after resection. Solid tumor tissue was carefully minced with scissors and isolated into small mixtures by pipetting. Appropriate amounts of delicate tumor tissue fragments were seeded into 25 cm^2^ cell culture flasks. The tumor cells were initially cultured in Opti-MEM medium supplemented with 5% heat-inactivated fetal bovine serum (FBS) (O5). Cultures were maintained in RPMI 1640 medium containing 10% heat-inactivated FBS (R10). Initial passages were completed when abundant tumor cell growth was observed, and consecutive passages were done every 1 or 2 weeks. During passaging, adherent cells were recovered by pipetting by treatment with trypsin while growth was subconfluent. Differential trypsinization was used to obtain a pure tumor cell population when stromal cell growth was noted in the initial cultures. Cultures were maintained in humidified incubators at 37 °C with 5% CO_2_ and 95% air. The established cell lines used in the present study are available at the Korea Cell Line Bank (https://cellbank.snu.ac.kr/). The cell lines were maintained in opti-DMEM medium supplemented with 20% fetal bovine serum (GIBCO).

### Cell proliferation assay

Cell proliferation assays were performed with EZ-Cytox (Daeillab Service) on cells seeded at 1 × 10^3^ cells/well in 96-well plates and cultured for the designated times. The absorbance of the plates was measured using a microplate reader (Molecular Devices) at a wavelength of 450 nm.

### Colony-forming assay

To grow colonies, cells were seeded in 6-well plates at 1000 cells/well density and incubated at 37 °C in an atmosphere of 5% CO_2_ for 14 days. The cell colonies were fixed and stained with 0.05% crystal violet-methanol-acetic acid solution after 14 days. Stained colonies were scanned and score was calculated.

### Western blot analysis

Western blot analysis was performed as previously described [10]. Antibodies against TGF-β (3711), Vimentin (5741), N-Cadherin (13,116), Claudin-1 (13,255), β-Catenin (8480), ZO-1 (8193), Snail (3879), Slug (9585), TCF/ZEB1 (3396), E-cadherin (3195), phosphorylated Smad2 at Ser465/467 (3108), Smad2 (5339), phosphorylated Smad3 at Ser423/425 (9520), Smad3 (9523), Smad4 (38,454) and ACTB (4967) were from Cell Signalling Technology; and HRP-conjugated IgGs (111-035-003 and 115-035-003) were from Jackson Immune Research. Immunoblots were visualized with a ChemiDoc XRS system (Bio-Rad). The density of the bands was measured using free image analyzer software (ImageJ V1.8x; National Institutes of Health, USA, http://rsb.info.nih.gov/ij/) [11].

### Telomere repeat amplification protocol (TRAP) assay with ELISA

A *TeloTAGGG* Telomerase PCR ELISA PLUS kit (Roche) was used according to the manufacturer’s protocol to measure the telomerase activity. GBM tissues or cells were homogenized in ice-cold lysis buffer using an automill (Tokken). Briefly, after BCA protein quantification of the lysates, 10 µg proteins were incubated in a total volume of 50 µl reaction mixture at 25 °C for 30 min to allow the telomerase to add telomeric repeats to the end of the biotin-labeled primer. Then, PCR was conducted for 33 cycles of 94 °C for 30 s, 50 °C for 30 s, and 72 °C for 90 s, followed by an additional extension time of 10 min at 72 °C and then holding the PCR tubes at 4 °C if not used immediately. The TA was measured at a reference wavelength of 450 and 690 nm. The relative TA (RTA) of each sample was calculated according to the instructions of the TeloTAGGG Telomerase PCR ELISA PLUS Kit.

### C-circle assay

C-circle detection was executed as previously described [12]. Briefly, 30 ng DNA was combined with 7.5 U Φ29 DNA polymerase (NEB), 0.2 mg/ml BSA, 1 mM each dATP, dGTP and dTTP, 10 μl 2X Φ29 buffer, 0.1% (v/v) Tween 20, and incubated at 30 °C for 4 h or 8 h followed by 20 min at 70 °C. Amplification products were transferred on a Hybond N + nylon membrane (Bio-Rad) and processed using the TeloTAGGG Telomere Length Assay Kit (Roche). Chemiluminescent signals were visualized with a ChemiDoc XRS system (Bio-Rad), and the intensity of the spots was quantified with ImageQuant TL software (Bio-Rad).

### In vivo xenograft tumor growth assay

To observe the in vivo proliferation of patient-derived primary GBM cells, a subcutaneous xenograft mouse model was constructed using 5-week-old female Balb/c nude mice. Primary GBM cells were cultured in Opti-MEM (LS31985070, Gibco) supplemented with 5% FBS (S 001-01, Welgene) and 1 × antibiotic–antimycotic (15240-062, Thermo Fisher Scientific) at 37 °C in a humidified incubator in the presence of 5% CO2. In total, 3 × 10^6^ cells were mixed with 100 µl of Opti-MEM and Matrigel mixture (Opti-MEM:Matrigel = 1:1) and subcutaneously injected into the mice. Tumor size was measured once a week using calipers, and tumor volume was calculated using the following formula: (length × width^2^)/2.

### Telomere length fragmentation assay

Telomere lengths were determined by Southern blot using a TeloTAGGG Telomere Length Assay Kit (Roche) according to the manufacturer’s protocol. Briefly, 1 µg of each DNA sample was digested with RsaI and HinfI for overnight at 37 °C, electrophoresed on a 0.8% agarose gel at 50 V for 4 h, and transferred to a nylon membrane by Southern blotting. The membrane was blocked and hybridized overnight to a digoxigenin (DIG)-labeled probe specific for telomeric repeats. Then, it was incubated with anti-DIG-alkaline phosphatase (1:1000 dilution) for 30 min and processed using the substrate in the TeloTAGGG Telomere Length Assay kit (Roche). After chemiluminescence signals were visualized with a ChemiDoc XRS system (Bio-Rad), telomere length was calculated with Telo Tool version 1.3.

### Whole exome sequencing and data analysis

Extracted DNAs from the samples were checked for quality control with Bioanalyzer. The sequencing library was prepared by random fragmentation of the DNA or cDNA followed by 5′ and 3′ adapter ligation. Library preparation was done using the SureSelectXT library prep kit. Whole exome sequencing (WES) was performed with the Illumina platform at Macrogen, Korea, and generated BCL binary was converted into raw FASTQ files utilizing the Illumina bcl2fastq package. Next FASTQ files were mapped to the reference genome (hg19) with BWA (0.7.12), and PCR duplicates were marked with Picard (1.130) [13]. Base recalibration and SNP and INDEL calling was done using GATK (v3.4.0) [14] We processed five different samples (blood, tumor, parent cell, subclone #5, and subclone #11) to obtain the WES data from the same patient. To track genomic alterations across the samples, the generated bam and vcf files were then analyzed with SuperFreq [15]. Typically, the SuperFreq program is designed for clonal analysis with samples related by timepoints. It tracks the SNP/INDEL and CNV from the samples and calls out clones or clusters according to the mutation changes. Since our samples do not contain any temporal information, we took advantage of the SuperFreq to track mutational differences and sample-specific mutations for both SNP/INDEL and CNV of our five samples. Mutation variations were clustered according to their variant allele frequency throughout the samples.

### RNA-sequencing and data analysis

Extracted RNAs from the samples were checked for quality control with Bioanalyzer. Library preparation was conducted using a Lexogen Quantseq 3’ mRNAseq library prep kit. Single-end mRNA sequencing was performed with Illumina Nextseq500 at ebiogen, Korea. Generated FASTQ files were mapped to the reference genome (hg19) with Bowtie2. Normalization of the count was done with EdgeR and batch effects were removed with the help of the R package limma (doi: 10.1093/nar/gks042 and 10.1093/nar/gkv007) [16, 17]. We then compared the general RNA-seq expression profile of the genes across the samples with Pearson correlation analysis using R. Differential gene expression (DEG) analysis between subclone #11 and #5 were done using the ExDEGA software provided by ebiogen Korea. Significant upregulated genes in subclone #11 compared to subclone #5 was selected with the filtering criteria of *P* value < 0.05 and Log2 fold change 11/5 ≥ 2. Then functional clustering of the upregulated genes in subclone #11 was done using DAVID v6.8 [18]. For the RNA-seq of GBM longitudinal samples, a total of 55 pairs (initial and recurrent) of tumor samples RNA was extracted and the library was prepared with a SureSelectV6 mRNA seq library preparation kit. RNA-sequencing was performed with Illumina Hiseq2500 at Macrogen, Korea. FASTQ files were processed and normalization of the count was done as described above. Then the differences between gene expression were calculated by subtracting the normalized count of each initial sample from its paired recurrent sample.

### Single cell RNA-seq analysis

We downloaded GBM single-cell RNA-seq data from four recently published studies (GSE117891, GSE131928, GSE125587, phs001287), which included a total of 71 samples from 65 patients [19–22]. Initially, we extracted the single-cell raw count data for each sample. We excluded the normal and immune cells, where necessary, based on the marker genes utilized in the original study data analyses. For selecting subclone #11 specific genes, we first extracted the genes located within subclone #11 specific chromosome 20 gain regions. For this, we started from a CNV LFC score table for each sample derived from SuperFreq analysis. Subsequently, we compared the CNV LFC values of the chromosome 20 gain region genes with at least 0.1 or higher CNV LFC differences between subclone #5 and #11. Then, we further filtered the genes according to the log fold change values (LFC ≥ 2, representing overexpression on subclone #11 samples) of RNA-seq DEG comparison of subclone #5 & #11. Through this process, a total of 9 genes (CDH4, CHRNA4, CPXM1, CTCFL, EEF1A2, PLCB1, RASSF2, SNORA51, STMN3) were selected (Fig. 4a). Additionally, we included the 5 EMT specific genes (GATA3, BMP4, ROBO2, GDNF, SOX11) that were upregulated in subclone #11 according to the RNA-seq pathway analysis to embrace the essential genes responsible for the phenotypic differences between subclone #5 and #11. We then analyzed the subclone #11 specific gene expression cumulatively and individually in the tumor cells of each GBM sample with Seurat version 3 [23]. Both the average gene expression levels for all of the candidate genes and the individual gene-level expressions per cell were plotted. In addition, we also analyzed the CNV status of samples harboring clusters similar to subclone #11 via inferCNV [24].

### Statistical analysis

The results were analyzed using IBM SPSS Statistics software (version 20.0; SPSS, Armonk, NY, USA). Data are expressed as the mean ± SE. Statistical significance was determined using Student’s *t* test. Kaplan–Meier curve analysis was used to analyze patient survival time. The differences with *P* values < 0.05 were considered statistically significant.

## Results

### Establishment of synchronous separate cell lines from the GBM tissue

The primary culture of GBM cells was performed from the freshly harvested cancer tissue from a 76-year-old female GBM patient. The radiological and histological characteristics of the original tumor showed typical features of GBM (Fig. S1a). A noteworthy radiological and gross finding was that the cortical direction of the tumor abutted upon the dura and disrupted the leptomeningeal membrane (Fig. S1b). The tumor was highly proliferative (Ki-67 index 68.7%) and showed positive glial fibrillary acidic protein and vimentin by immunohistochemistry (Fig. S1c–S1e). Its genetic features include wild-type IDH1/2, a TERT promoter mutation (C250T), and wild-type ATRX. Chromosomal abnormalities of Epidermal growth factor receptor (EGFR) gene amplification and chromosome 9p21/p16 locus deletion were revealed by fluorescence in situ (S1f, S1g).

**Fig. 1:**
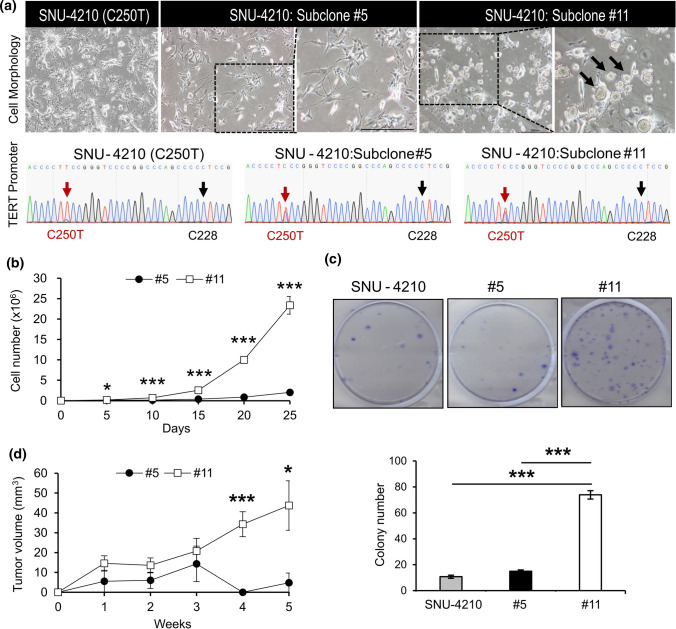
Cellular features of subclones #5 and #11 derived from SNU4210. **a** Subclone #5 and #11 derived from SNU-4210 exhibited different cell morphology. Subclone #5 grows in a fibroblast form and in subclone #11, spheroid formation is observed (black arrows). The genotypes of C228 and C250 of the TERT promoter were identified by Sanger sequencing in SNU-4210, subclone #5 and #11, respectively. **b** Cell proliferation assay of subclone #5 and #11 for 25 days. Cell numbers of subclone #11 were 11.5-fold higher than subclone#5 on day 25. **c** Colony-formation assays with SNU-4210, subclone #5 and #11. **d** Subcutaneous injection in BALB/c nude mice with subclone #5 and #11 (*n* = 5). Tumor size was measured for 5 weeks. Consistent with the results of the in vitro experiments, the size of the tumor formed from subclone #11 increased faster than in subclone #5. Data and error bars show mean ± sd of three independent biological replicates (*n* = 3). *P* values were obtained using the two-tailed Student’s *t* test. *P < 0.05, ***P < 0.001

Cells were dissociated from the freshly resected primary tumor tissue shortly after the surgery and primary cell lines were established as described previously [25]. Successful establishment of the cell line was confirmed after successive subculture and quality assessment, and the cell line was named SNU-4210 (Fig. 1a). During the subculture passages, we discovered the two kinds of cells with distinct morphology in the same dish. We carefully isolated cells of different shapes and then repeatedly isolated colonies derived from single cells of each form to separate them completely. Careful isolation of the cells with different shapes and subculture identified the same *TERT* promoter mutation in both cells, implying they were both cancer cells descended from a common ancestor (Fig. 1a). The daughter cell lines were named SNU-4210 #5 (subclone #5) and SNU-4210 #11 (subclone #11) after their original passage numbers. The subclone #5 showed a fibroblast-like shape, whereas subclone #11 grew with spheroid formation. Next, we compared the biological characteristics of subclone #5 and subclone #11. Cell proliferation and cell viability assays showed that subclone #11 had a significantly higher proliferative activity than subclone #5 (Fig. 1b). The colony-forming assay also showed an increase in colony formation by subclone #11 as compared to subclone #5 (Fig. 1c). In vivo experiments through subcutaneous injection into BALB/c nude mice showed that subclone #11 had a higher growth potential compared with subclone #5 (Fig. 1d).

**Fig. 2:**
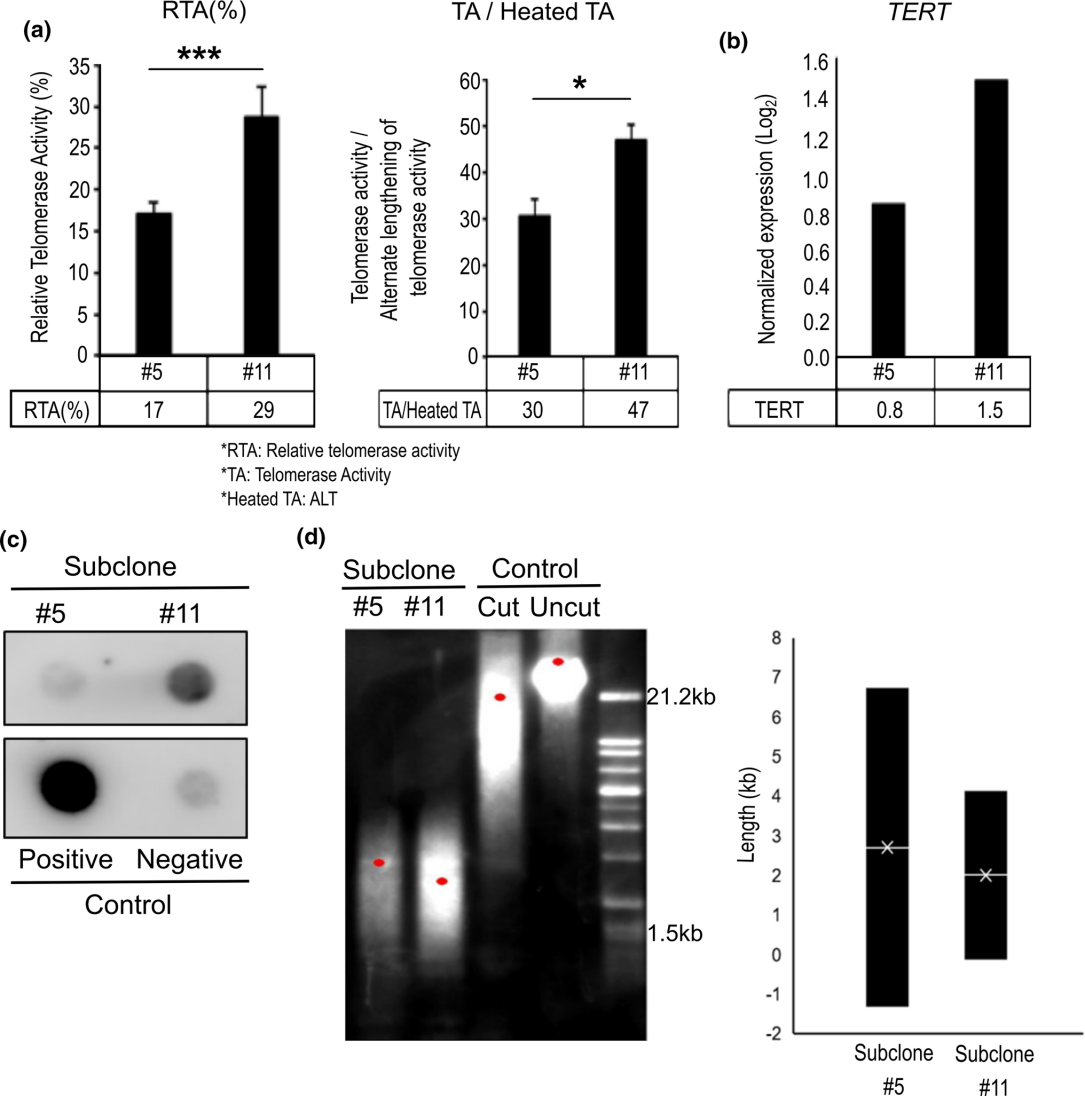
Comparison of telomerase activity and telomere length in patient-derived glioblastoma. **a** Trap assay showing that both telomerase activity (left panel) and alternative lengthening of telomeres (ALT) activity (right panel) were higher in subclone #11. **b** TERT gene expression of subclone #11 was also comparatively higher than subclone #5. **c** C-circle analysis also confirmed that ALT activity was more active in subclone#11 than subclone #5. **d** Telomere restriction fragment (TRF) analysis showed that the telomere length of subclone #11 was shorter than in subclone#5

While both subclones consistently maintained the same *TERT* promoter mutation (C250T) of the original tumor and cell line, subclone #11 showed higher enzymatic activity of telomerase and *TERT* expression compared with subclone #5 (Fig. 2a, 2b). Moreover, evidence of alternative lengthening of telomeres (ALT) measured by c-circle assays was found in subclone #11, which was not evident in subclone #5 (Fig. 2c). Telomere restriction fragment (TRF) analysis revealed that the telomere length of subclone #11 was shorter than subclone #5 and the telomere length range of subclone #11 was less than subclone #5, indicating less telomere length heterozygosity (Fig. 2d). These results show that subclone #5 and #11 derived from SNU-4210 have distinctly different biological characteristics, suggesting more oncogenic aggressiveness of subclone #11.

### Clonal evolution of the subclones analyzed by genomic composition

**Fig. 3:**
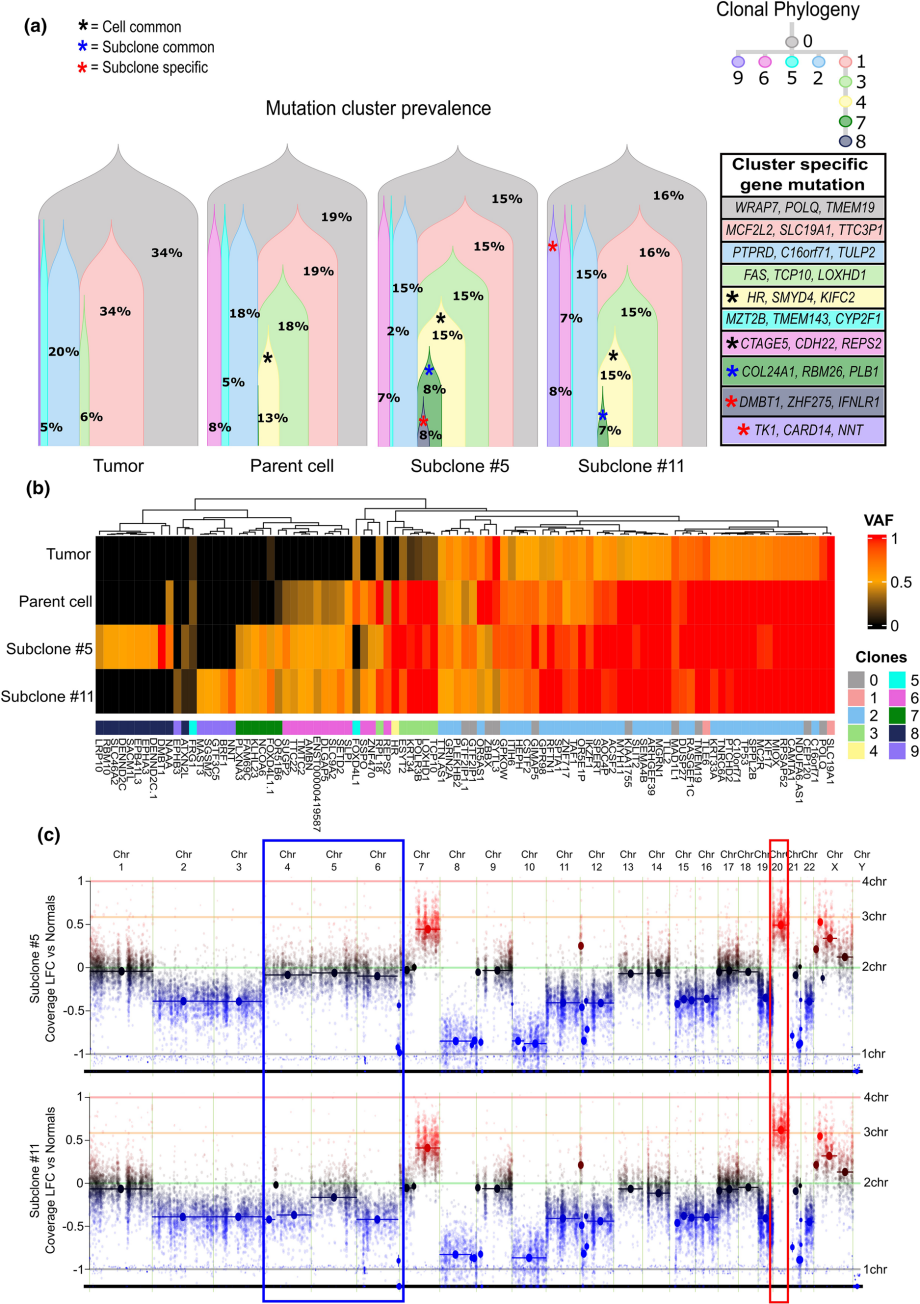
Whole exome analysis. **a** Tracking genomic alterations (somatic mutations and CNV) changes from tumor to parent cell to subclone #5 and #11 by clonal phylogeny cluster analysis. Each of the subclones showing specific clusters, Cluster 8 for subclone #5 and cluster 9 for subclone #11. **b** Heatmap showing amino acid changing somatic mutations that are retained or newly acquired from tumor to parent cell to subclone #5 and #11. No GBM significant genes were present among the subclone-specific mutations. **c** CNV changes in #5 and #11 showing copy number loss in chromosomes 4, 5 and 6 within the blue rectangle and copy number gains in chromosome 20 within the red rectangle when comparing the copy number of subclone #5 to subclone#11

We analyzed and compared the somatic mutation and copy number variation (CNV) profile in the SNU-4210 cell line with its subclones and the original GBM tissue using whole-exome sequencing (WES) data. To track the clonality across the tissue sample and cell lines with temporal information, we took advantage of the SuperFreq algorithm to cluster the mutational changes according to the prevalence of their variant allele frequency. A total of 10 clusters across the samples were identified (Fig. 3a). The common cluster with the highest prevalence in all of the samples was detected as the germline cluster, and 4 clusters (clusters 1, 2, 3, and 5) were conserved across the tissue sample to the cell lines and its subclones (Fig. 3b). Among the other newly developed 5 clusters in the cell lines, 2 clusters (cluster 4 and 6) were common, while cluster 7 was specific to both subclones (Fig. 3b). The clusters 8 and 9 were specific clusters to subclone #5 and #11, respectively (Fig. 3b).

When we analyzed the subclone-specific mutations, none of those mutations were found to reside in any of the previously reported GBM driver genes (Fig. 3c). The full list of cluster specific mutations is included in supplementary Table S1. However, when we analyzed the CNV status, noticeable copy number loss regions in Chromosome 4, 5 and 6 and a gain in Chromosome 20 were identified in subclone #11 (Fig. 3c). These findings support the impact of intratumoral heterogeneity on the clonal evolution process during disease progression in GBM.

### Emergence of aggressive subclones accompanied by EMT signature

**Fig. 4:**
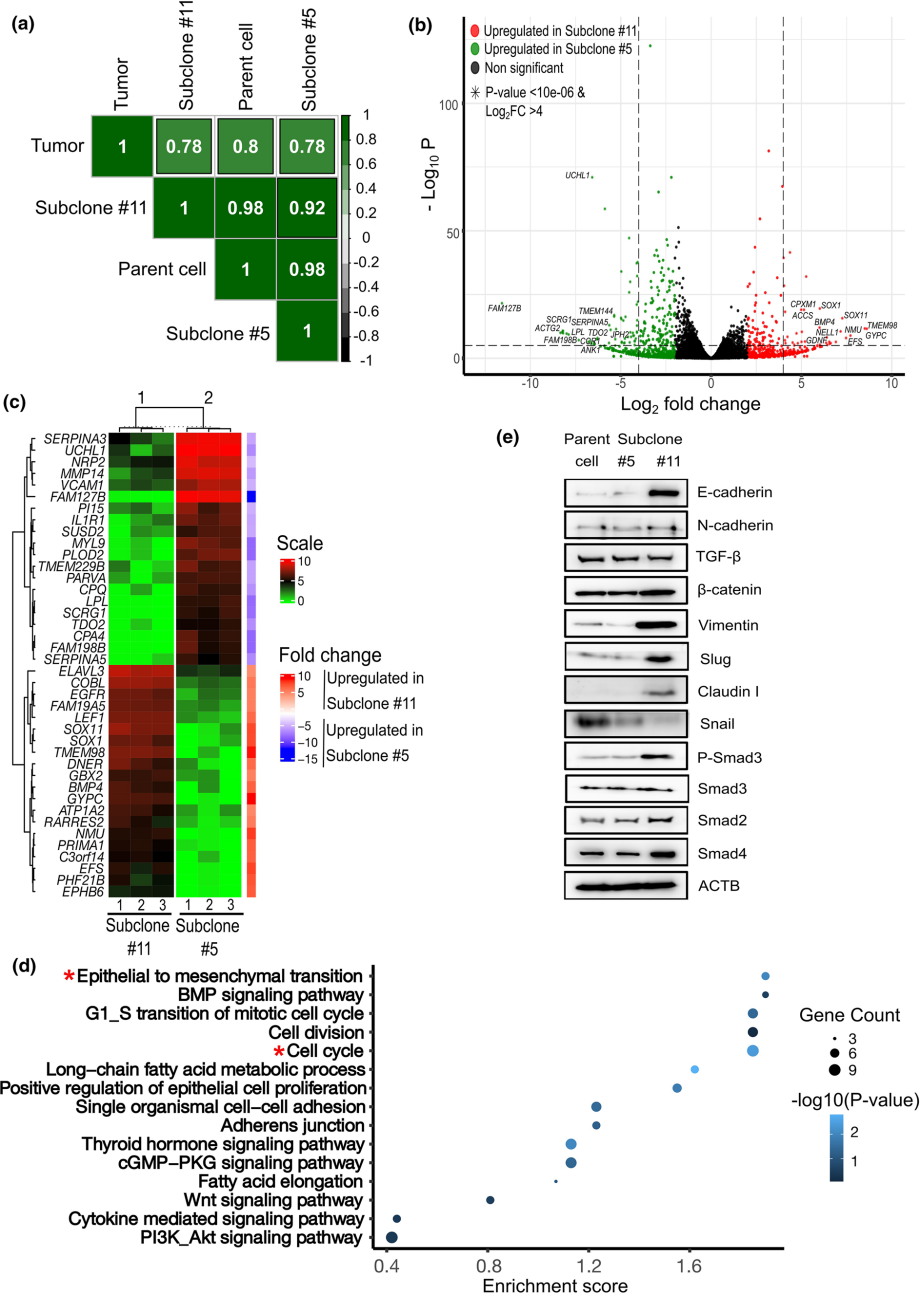
RNA-seq analysis. **a** Pearson correlation of the overall expression profile showing minimal differences between subclone #5 and #11 with the parent tissue and cells. The numbers in the boxes represent the correlation coefficient. Each subclone has expression correlated 78% and 98% with the parent tissue and cells, respectively. The correlation between the two subclones was 92%. **b** Volcano plot for identifying differentially expressed genes in subclone #5 and #11 showing significant amounts of DEGs between the two subclones despite similar overall gene expression. **c** Heatmap of the top 20 differentially expressed genes between subclone #5 (group 2) and #11 (group 1). **d** Functional annotations of significantly upregulated genes in subclone #11 compared to subclone#5 (FC ≥ 2, *P* value < 0.05) showing the EMT and cell cycle-related gene ontology clusters being significantly upregulated in subclone #11 (ES > 1.5, *P* value < 0.05). **e** Western blot analysis for EMT-related protein expression in SNU-4210, subclone #5, and #11 showing higher expression of E-cadherin, vimentin and slug in subclone #11 compared to #5

Despite the distinct genetic differences, the overall gene expression profiles displayed similarity between subclone #5 and #11 (Table S2). Pearson correlation analysis of the overall gene expression profile of the subclones showed a 78% positive correlation with the parent tumor, a 98% positive correlation with the parent cell line, and a 92% positive correlation with each other (Fig. 4a). However, we were also able to discover significant differentially expressed genes (DEGs) between these subclones (Fig. 4b, c). When we matched the DEGs and CNV between the subclones, we could confirm that the genes within the regions displaying copy number loss in chromosomes 4, 5, and 6 for subclone #11 were significantly downregulated (Log2FC < -2, *P* value < 0.05), and genes within the regions showing copy number gains in chromosome 20 for subclone #11 were upregulated (Log2FC > 2, *P* value < 0.05) compared to subclone #5 (Table S3). Functional clustering of upregulated genes in subclone #11 compared to subclone #5 showed significant enrichment on EMT and cell cycle-related gene ontology clusters (Enrichment score > 1.5, *P* value < 0.05) in subclone #11 (Fig. 4d). We could confirm that the major EMT-related genes were overexpressed in subclone #11 compared with subclone#5 at the protein level (Fig. 4e). These results show that the aggressiveness of subclone #11 can be explained by the emergence of mesenchymal transition.

### Presence of subclone#11 like cell clusters in GBM

**Fig. 5:**
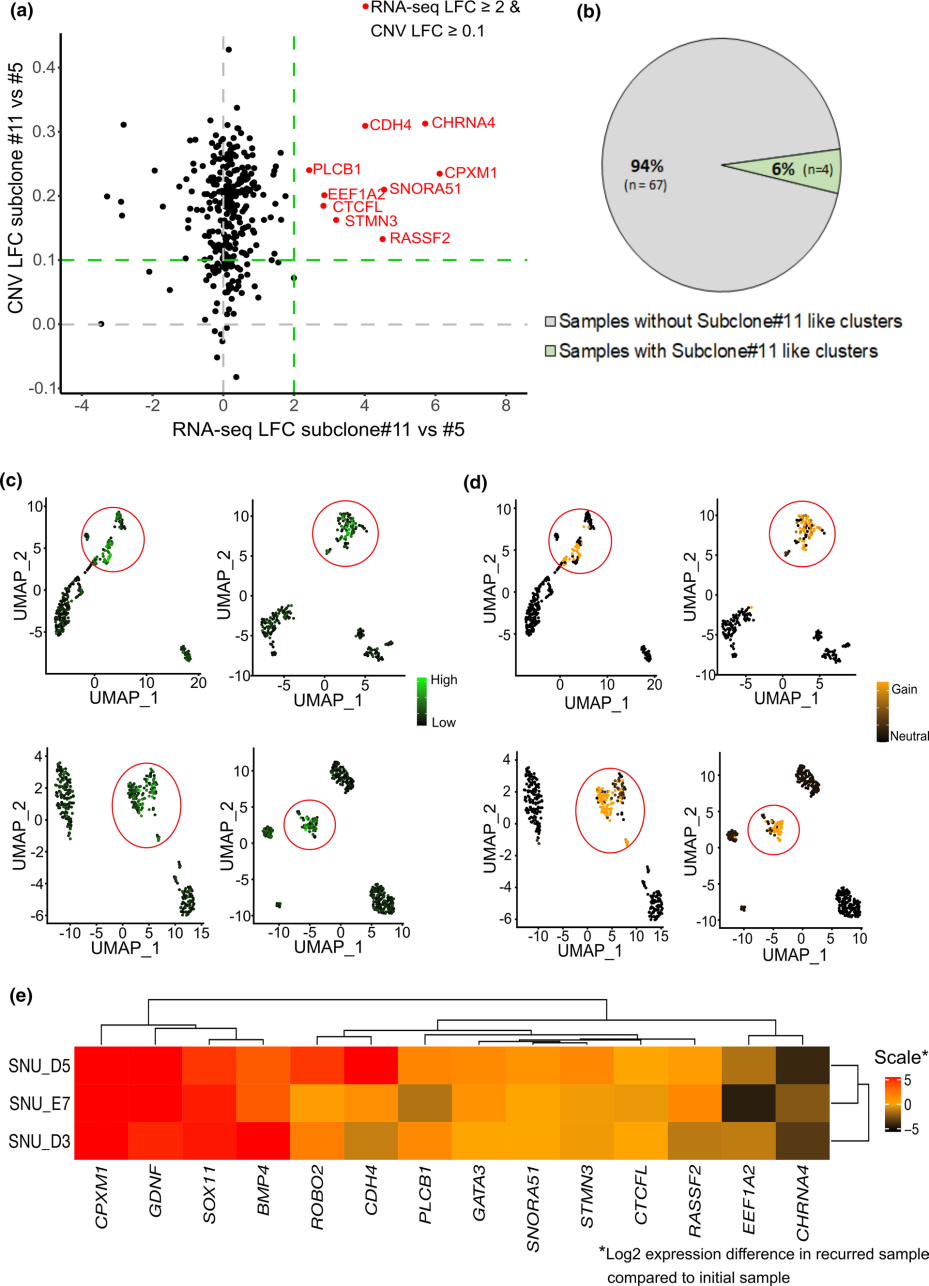
Evidence of subclone #11 like clusters in single-cell RNA-seq data and their role in GBM progression in GBM longitudinal data. **a** Subclone #11 specific gene selection based on RNA-seq log fold change and CNV log fold change inside chromosome 20. Genes with RNA-seq LFC > 2 and CNV LFC > 0.1 were selected as subclone #11 specific genes. **b** A summary of glioblastoma single-cell RNA-seq data analysis for the identification of the subclone #11 specific gene enriched cluster. Three recent GBM single-cell RNA-seq studies were subjected to the analysis and 4 samples out of 71 (6%) harbored subclone #11 specific gene enriched cluster. **c** UMAP clustering results with colors representing the average level of subclone #11 specific gene expressions in samples harboring subclone #11 specific gene enriched clusters. **d** UMAP clustering results with colors representing the level of subclone #11 specific Chr 20 amplification for the samples containing subclone #11 specific gene enriched clusters. **e** Gene expression heatmap corresponding to the individuals displaying overexpression of selected genes of subclone #11 in recurrent samples compared to the initial samples

Based on previous observations that identified mesenchymal-related molecular signatures for GBM tumors and stem cells [26], and our results showing a mesenchymal like phenotype for subclone #11, we posited that there might be other GBM tumors harboring subclone clusters similar to subclone #11 within the tumor. For this, we selected 14 subclone #11 specific genes, of which 9 genes had both a higher copy number (LFC ≥ 0.1) and a gene expression level (LFC ≥ 2) for subclone #11 comparing to subclone #5 (Fig. 5a and Table S4) and five from the EMT-related pathways (the selection criteria details are described in the methods). To explore whether any similar patterns could be observed in other GBM tumor samples, we analyzed the publicly available single-cell RNA-seq expression profiles from GBM tumors, explicitly focusing on these 14 subclone #11 specific genes. For this, we analyzed a total of 71 GBM samples from four different single-cell RNA-seq studies (Fig. S2, S3, S4, S5). Among these samples, we found four samples that had subclone #11 like clusters (Fig. 5b). These distinct clusters showed overexpression of the subclone #11 specific genes, and all four samples showed chromosome 20 amplification in these clusters (Fig. 5c, d, S6). These results imply that the intratumor heterogeneity harboring subclone #11-like cells is present in at least a subset of GBM patients.

### Upregulation of subclone#11 gene signatures in recurrent GBM samples

Next, we investigated if the molecular features harbored in subclone #11 were linked to GBM progression and recurrence. To validate our hypothesis, we analyzed the RNA-seq data of a set of longitudinal GBM samples (methods). We first extracted the normalized gene expression values of the Hallmark EMT gene set from the MSigDB database (https://www.gsea-msigdb.org/gsea/msigdb/) for all of the paired longitudinal samples and then calculated the gene expression difference between the recurrent and initial samples. We found 10 pairs that showed significantly high expression for most of the EMT genes in the recurrent samples, suggesting that the mesenchymal transition process was the key factor driving the recurrence of these pairs. We then investigated the same subclone #11 specific genes that we used for the scRNA-seq analysis to see if any of these 10 paired samples had upregulation of the subclone #11 specific genes. Indeed, we were able to find 3 samples among the 10 displaying overexpression of a subset of subclone #11 specific genes (Fig. 5e). These findings provide evidence that subclone #11 like cells in GBMs contribute to the intertumoral heterogeneity and are also linked to the progression and recurrence of GBM due to its EMT-like characteristics.

## Discussion

Despite a significant amount of research on the oncogenesis and progression of GBM, the establishment of a breakthrough anti-cancer treatment strategy is still a long way off. The invasiveness and heterogeneity nature of GBM are major hurdles to overcome for the development of a successful treatment strategy. The acquisition of mesenchymal transition during the course of GBM progression has been reported to be one of the mechanisms driving intratumoral heterogeneity followed by treatment resistance [27]. However, data linking mesenchymal transition and intratumoral heterogeneity in clinical samples of GBM are still inadequate to establish therapeutic strategies targeting mesenchymal transition in GBM. In the present study, we provided evidence of a mesenchymal transition process linked to intratumoral heterogeneity in a subset of cells within the same clinical sample and investigated the significance of the findings using several other GBM clinical samples. For this, we conducted a streamlined characterization of intratumor heterogeneity of an actual GBM case from the isolation of subclones and their subsequent molecular and phenotypic characterization. We found that one of the subclones had upregulation of EMT pathways through genetic analysis and we could validate these findings in its protein expression levels. Morphologically, this subclone showed more stem-cell-like characteristics and it engaged in spheroid formation in culture; in previously published research, it was reported that cells undergoing EMT most likely acquire stem-cell-like properties [28, 29]. After successfully establishing two discrete synchronous cell lines derived from the same GBM tissue, we identified distinct genetic features that characterize each cell line. We also demonstrated that the subcellular evolution of mesenchymal transition in one subclone could be simulated in a disease progression scenario through comparison with longitudinal data.

Inter- and intratumoral heterogeneity of GBM has been identified for a number of years through transcriptomics, genomics and single-cell transcriptomics [6, 20, 30]. We could simulate the development of aggressive oncogenicity in one of the subclones in relation to the emergence of the mesenchymal transition process. We also confirmed changes in the telomere maintenance mechanism between the two subclones, and subclone #11 showed the usage of an ALT-mediated telomere lengthening mechanism. ALT has been frequently associated with mesenchymal and stem cell origins, which cells are also closely associated with EMT phenomenon [31, 32].

Previously, multiple cell lines derived from the same tumor samples have been used to study the course of progression and metastasis of the tumor in lung, breast and prostate cancers [33–35]. These studies mainly focused on establishing the cell line and characterizing them in the context of the progression of the cancer. However, identifying two morphologically different cell types from the same primary tissue and subsequent establishment of two different cell lines are not easy. Moreover, thorough characterization of these synchronous cell lines, which has not previously been comprehensively performed, can contribute to further research on intratumoral heterogeneity and its oncological behavior.

To confirm the reproducibility of occult clones and subcellular evolution of mesenchymal transition in GBM, we looked for tumors displaying subclone #11-like characters via re-analyzing publicly available single-cell RNA-seq data. We could successfully find four tumor samples containing such clusters. Although samples from only one study [19] out of four demonstrated subclone clusters similar to subclone #11, this might be due to the differences in sampling methods, where Yu et al. conducted multi-sector biopsies taken from each tumor, which takes spatial differences inside a tumor into consideration. Our result further emphasizes the need for multi-sampling of a tumor to better characterize its intratumor heterogeneity and to identify major clusters or subclones inside a tumor. We could also validate that not only that cell clusters like subclone #11 can contribute to intratumoral heterogeneity in GBMs but it also could drive the recurrence of the tumor owing to the activation of mesenchymal transition. These findings could serve as a good example of bed to bench side work by not only characterizing intratumoral heterogeneity per se but also establishing a cellular model, a resource that can be used for further investigation of intratumoral heterogeneity and the mesenchymal transition process.

The progression of mesenchymal transition through upregulation of the EMT pathway in one of the subclones is one of the crucial findings of our study. We showed that this type of EMT activated subclone is responsible for some of the intratumoral heterogeneity in GBMs. The mesenchymal transition process has been well established as one of the leading causes of intratumoral heterogeneity in multiple studies [8, 36]. Mesenchymal transition has also been described as an important process that drives GBM progression in a recent study [37]. In addition, the mesenchymal transition process in GBMs has been said to be triggered by various causes like a hypoxic microenvironment, EMT inducing signals (Twist, Snail, Slug and ZEB), microRNAs, prolonged radiation and anti-VEGF therapy [27]. Activation of mesenchymal transition in GBMs leads to more aggressiveness and recurrence of the tumor, as we have also found in our study.

## Conclusions

Our study describes direct evidence of the significance of mesenchymal transition activation in GBM contributing intratumoral heterogeneity and tumor recurrence. Synchronous cell lines derived from the same clinical samples that exhibit a heterogeneous character can shed new light on studying the role of mesenchymal transitions in GBMs and provide valuable knowledge for developing treatments targeting the complex characteristics of GBM.

## Supplementary Information

Below is the link to the electronic supplementary material.

Supplementary file1 (DOCX 5639 kb)

## Data Availability

Data and materials for the study are included in the manuscript and supplementary information.

## Abbreviations

GBM: Glioblastomas
EMT: Epithelial–mesenchymal transition
MET: Mesenchymal to epithelial transition
EGFR: Epidermal growth factor receptor
ALT: Alternative lengthening of telomeres
TRF: Telomere restriction fragment
CNV: Copy number variation
WES: Whole-exome sequencing
DEGs: Differentially expressed genes
