# Supplementary material for: Subcellular progression of mesenchymal transition identified by two discrete synchronous cell lines derived from the same glioblastoma
Source: Cell Mol Life Sci. 2022 Mar 12;79(3):181. doi: 10.1007/s00018-022-04188-3 (PMC8918182; doi:10.1007/s00018-022-04188-3)
Supplement: Supplementary file 1 — Supplementary file1 (DOCX 5639 kb) [file 18_2022_4188_MOESM1_ESM.docx]

**Additional file 1**

**Supplementary Information:**

**Subcellular progression of epithelial-mesenchymal transition identified by two discrete synchronous cell lines derived from the same glioblastoma**

**Supplementary Figures**

Figure S1. A case of 76-year-old glioblastoma (GBM) of which the origin of cell lines.

Figure S2. Subclone #11 specific genes expression in patient samples of Yu et al (2020) study.

Figure S3. Subclone #11 specific genes expression in patient samples of Neftel et al (2019) study.

Figure S4. Subclone #11 specific genes expression in patient samples of Xiao et al (2019) study.

Figure S5. Subclone #11 specific genes expression in patient samples of Wang et al (2021) study.

Figure S6. Individual expression profile of highly expressed subclone #11 specific genes in four positive samples.

**Supplementary Tables**

Table S1. Details of SNP mutations and CNV regions unique to subclone #5 and #11.

Table S2. Overall gene expression of SNU-4210 parent tissue, cell and subclone #5 & #11.

Table S3. DEG values of genes of the significant CNV regions between subclone #5 and #11.

Table S4. Selection of subclone #11 specific genes comparing mRNA expression and CNV region expression.


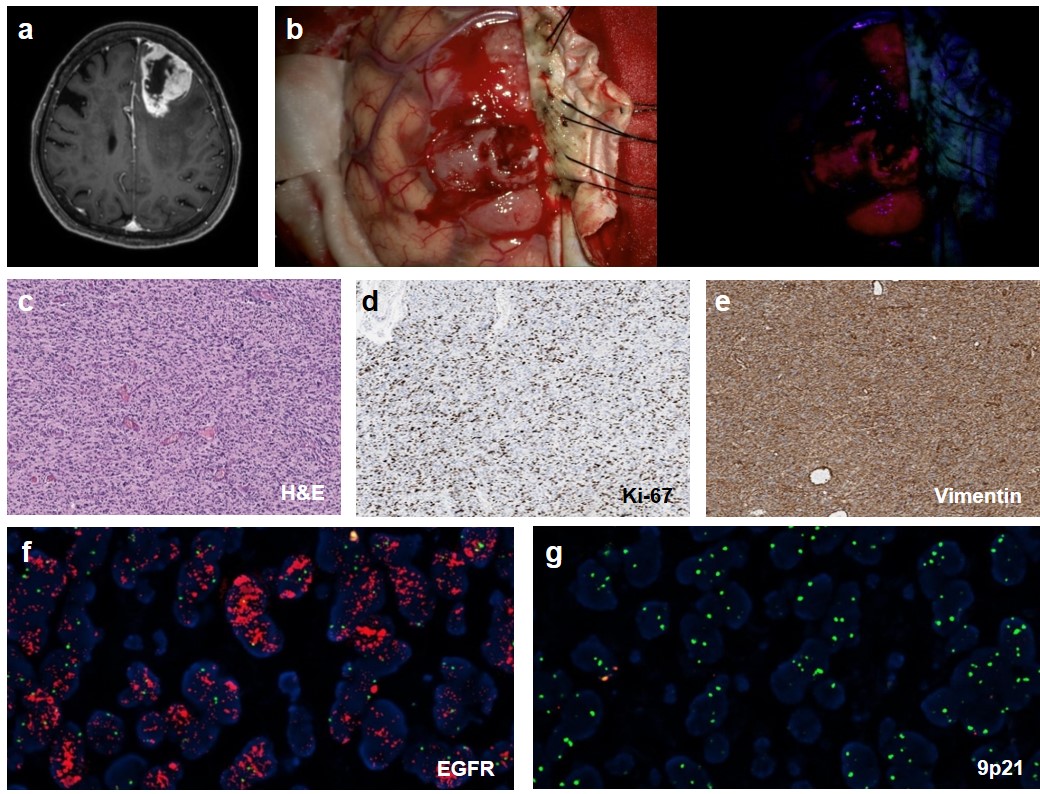


**Figure S1. A case of 76-year-old glioblastoma (GBM) of which the origin of cell lines.** **a.** Magnetic resonance images show the well-enhancing mass in the left frontal lobe with central necrosis. **b.** Surgical view shows tumor mass intruding leptomeningeal membrane abutting on dura (left). Fluorescence-guided surgery with 5-aminolevulinic acid exhibits strong red fluorescence in tumor mass usual for GBM. **c.** Microscopic features with typical GBM (H&E bar: 200 μm). **d.** High Ki-67 labeling index of 68.7% (bar: 200 μm). **e.** Immunohistochemical strong positivity for vimentin (bar: 200 μm). **f.** Epidermal growth factor receptor (EGFR) gene amplification by fluorescence in situ hybridization (FISH). **g.** Homozygous chromosome 9p21/p16 locus deletion by FISH. Absence of red signals in most of the nuclei and retained green signals for CEP9.


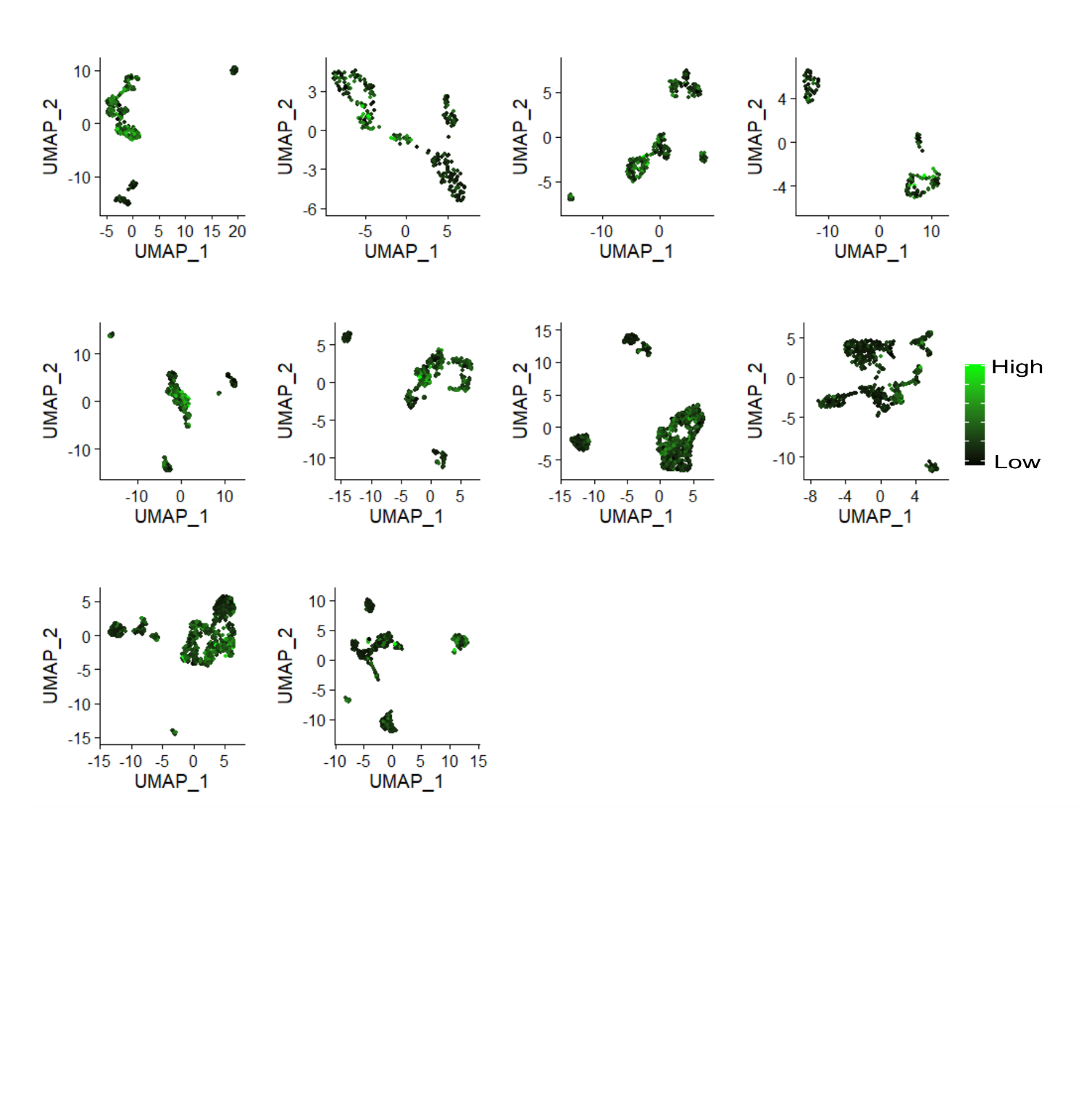


**Figure S2. Subclone #11 specific genes expression levels for patient samples from Yu et al (2020) study.** 4 samples from this cohort (Fig 4c) out of 14 did show any specific clusters enriched for expression levels of subclone #11 specific genes.


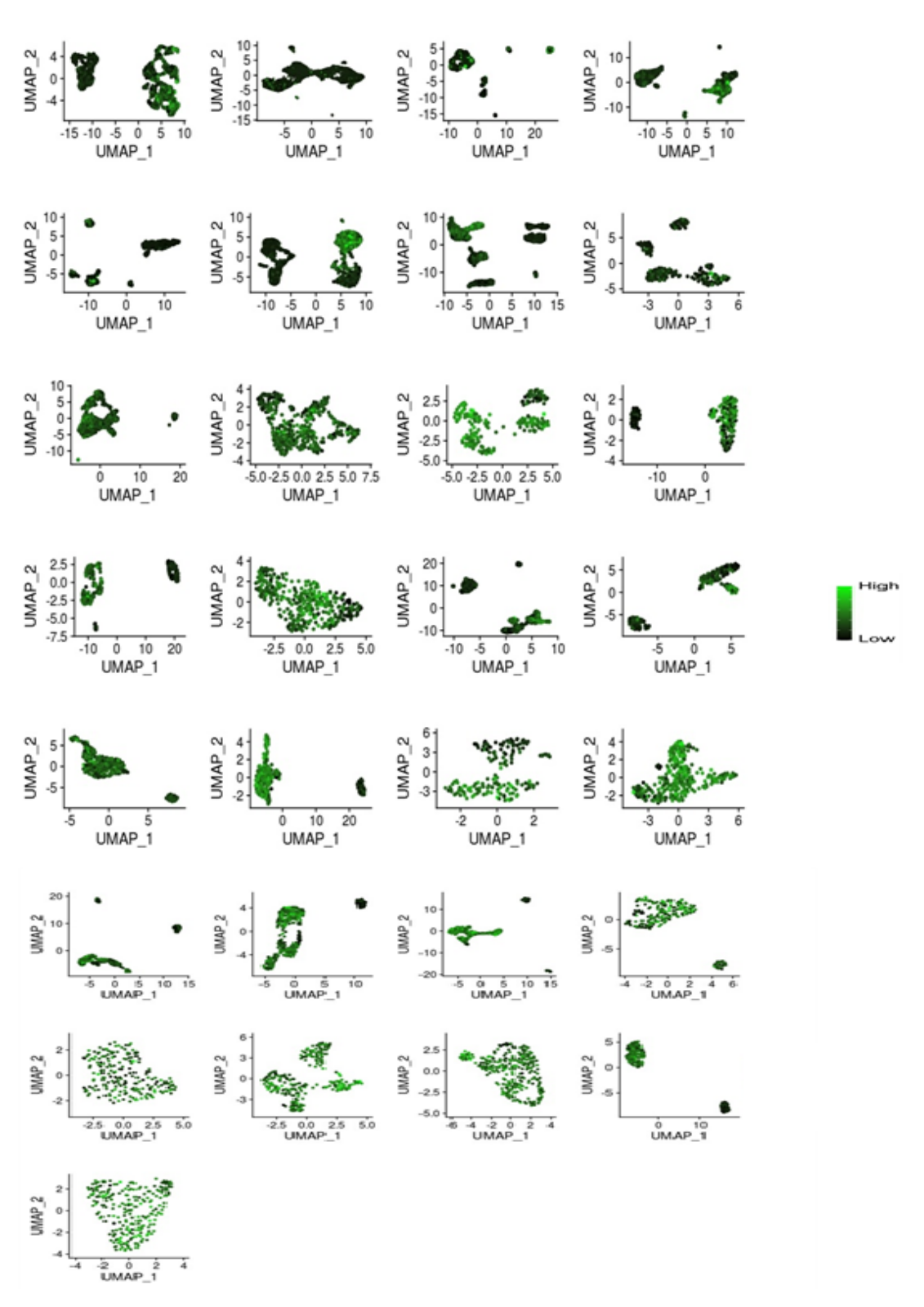


**Figure S3.** **Subclone #11 specific genes expression levels for patient samples from Neftel et al (2019) study.** All 29 samples subjected to the analysis did not show any specific clusters enriched for expression levels of subclone #11 specific genes.


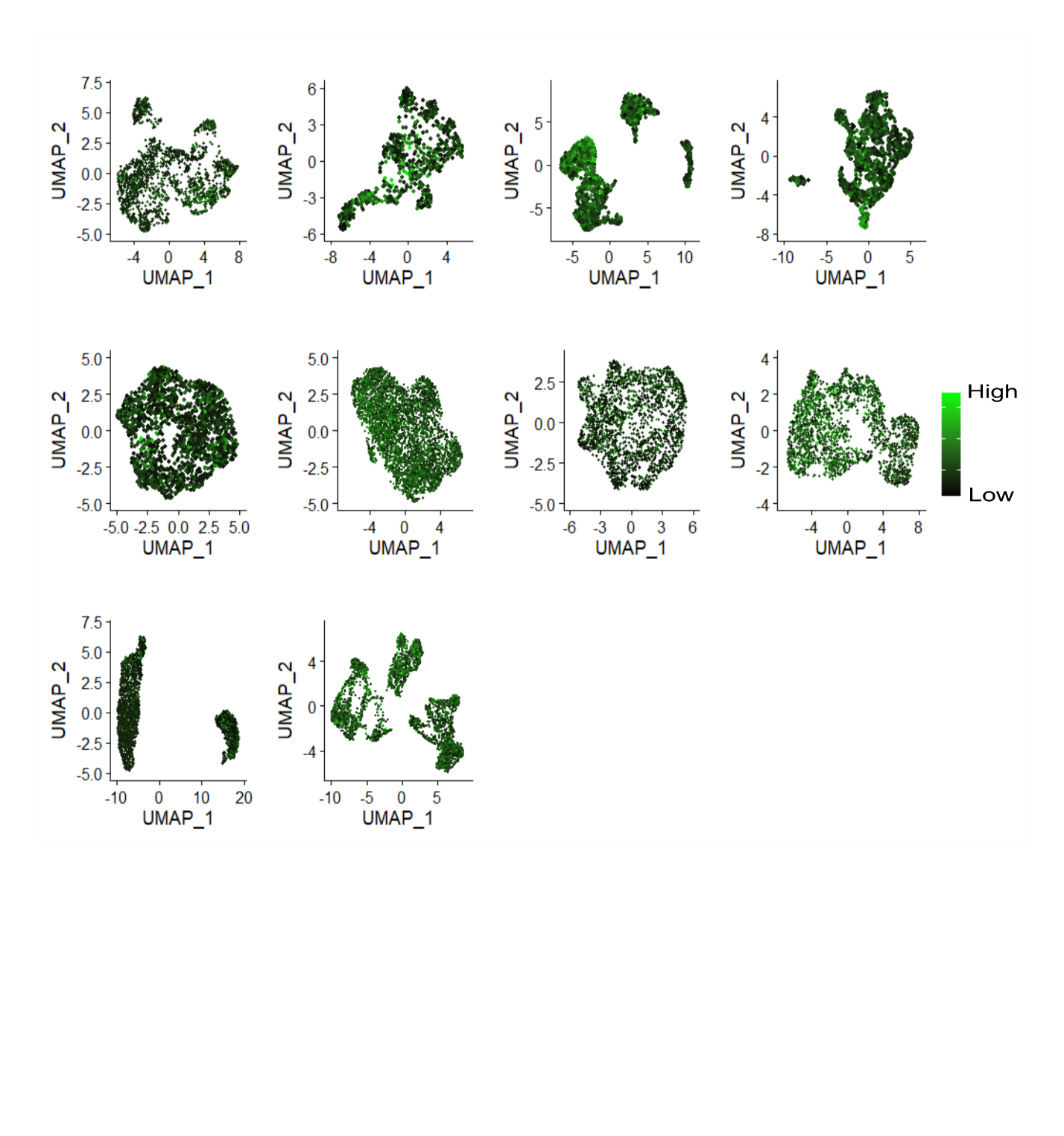


**Figure S4. Subclone #11 specific genes expression levels for patient samples from Xiao et al (2019) study.** All 10 samples subjected to the analysis did not show any specific clusters enriched for expression levels of subclone #11 specific genes.


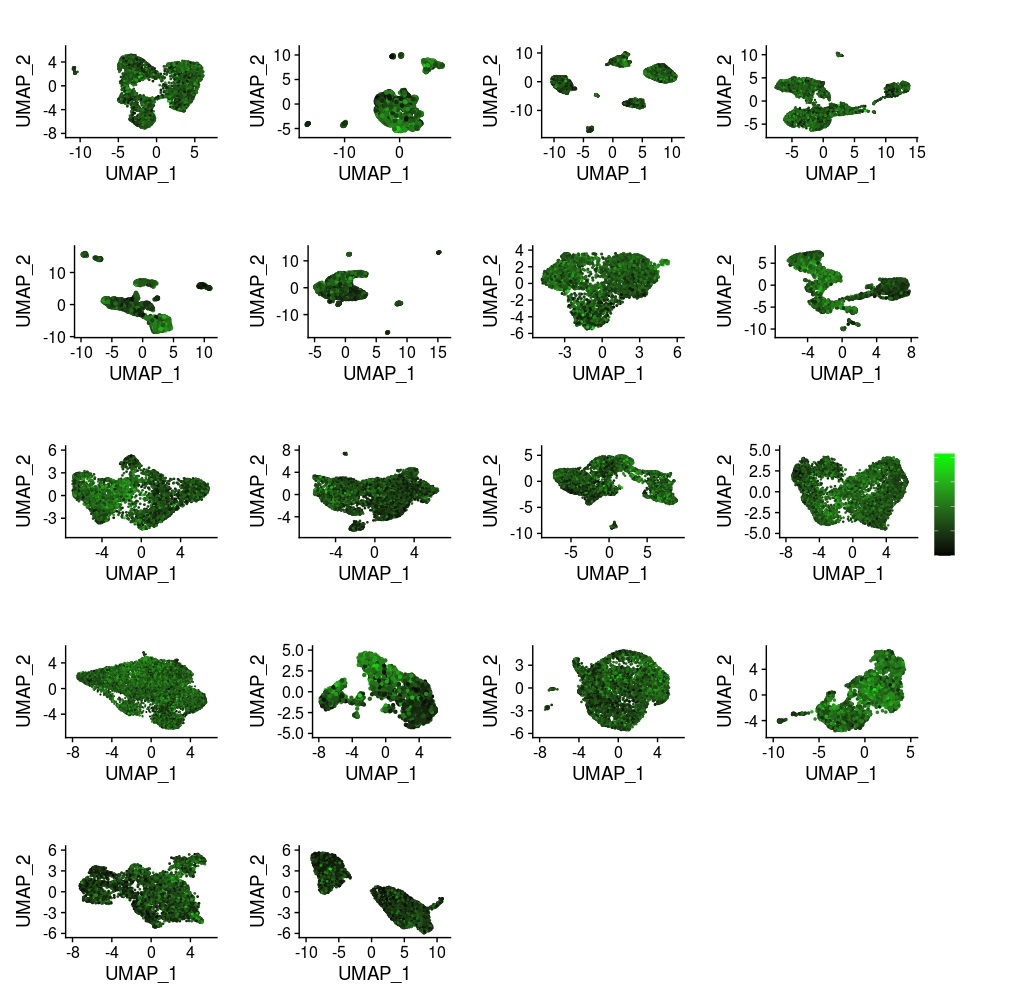


**Figure S5. Subclone #11 specific genes expression levels for patient samples from Wang et al (2021) study.** All 18 samples subjected to the analysis did not show any specific clusters enriched for expression levels of subclone #11 specific genes.


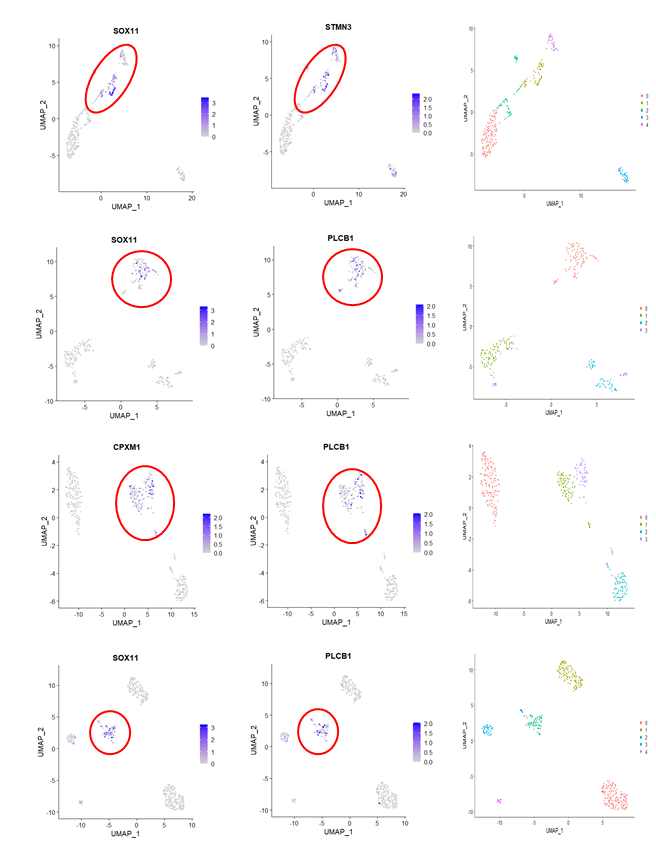


**Figure S6.** **Individual expression profile of highly expressed subclone #11 specific genes in four positive samples.** Top 2 highly expressed genes (*BMP4, SOX11*) among the subclone #11 specific genes were shown in four positive samples. Each sample cell clusters are also shown on the far right of each row for comparison.

**Table S1. Details of SNP mutations and CNV regions unique to subclone #5 and #11.**

**Table S2. Overall gene expression of SNU-4210 parent tissue, cell and subclone #5 & #11.**

**Table S3. DEG values of genes of the significant CNV regions between subclone #5 and #11.**

**
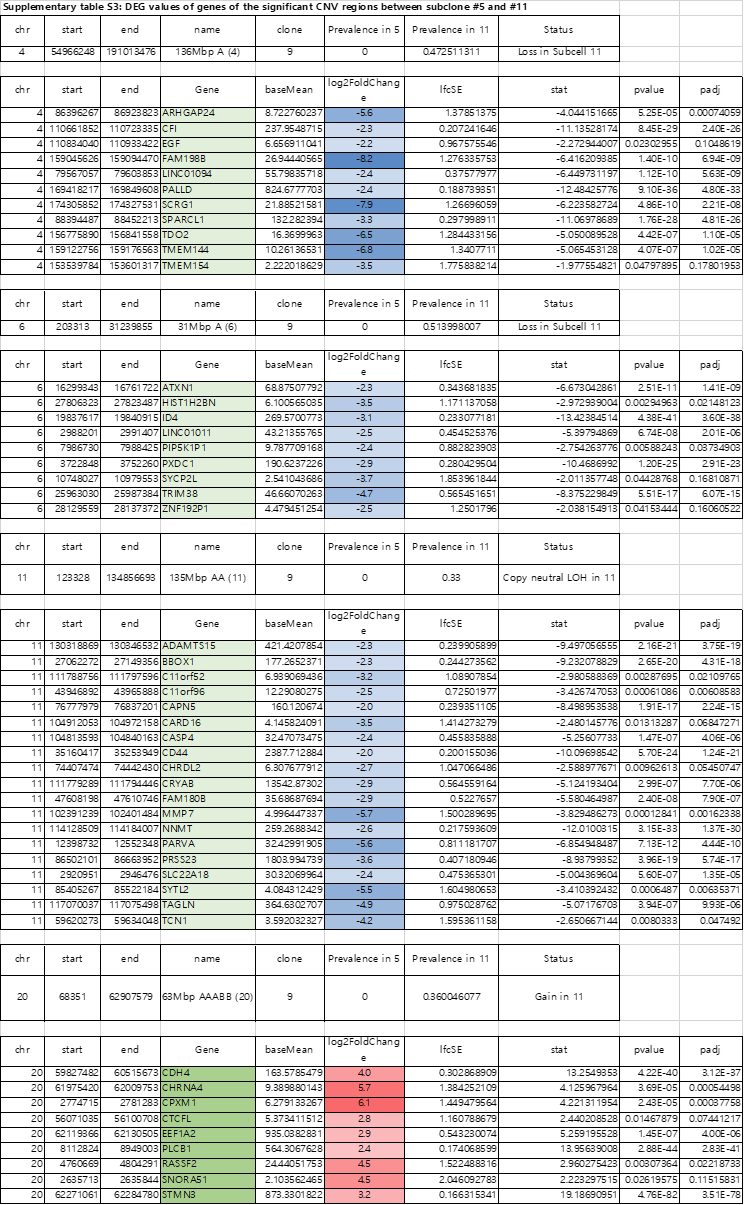
**

**Table S4. Selection of subclone #11 specific genes comparing mRNA expression and CNV region expression.**
